# Eosinophilic Fasciitis in Children: A Case Report

**DOI:** 10.7759/cureus.114611

**Published:** 2026-08-16

**Authors:** Karima El Fakiri, Mohamed Zamalik, Noureddine Rada, Mohammed Bouskraoui

**Affiliations:** 1 Pediatric A Department, Mohammed VI University Hospital, Marrakech, MAR; 2 Faculty of Medicine and Pharmacy, University Cadi Ayyad, Marrakech, MAR

**Keywords:** child, cutaneous abscess, eosinophilia, eosinophilic fasciitis, shulman’s disease, skin and fascial biopsy

## Abstract

Eosinophilic fasciitis (EF), also known as Shulman’s disease, is a rare inflammatory and fibrosing disorder characterized by inflammation and eosinophilic infiltration of the fascia, leading to skin thickening and induration. It typically presents with limb edema progressing to induration and sclerosis and is frequently, but not invariably, associated with peripheral blood eosinophilia. Diagnosis relies on a combination of clinical, laboratory, imaging, and histopathological findings. We report the case of a 16-year-old female patient with a documented history of hypereosinophilic syndrome, moderate persistent asthma, and recurrent cutaneous abscesses, who presented with localized swelling of the left leg approximately one week after minor trauma. Clinical examination revealed a 3-cm fluctuant swelling with local inflammatory signs. Laboratory investigations showed a negative C-reactive protein level and an eosinophil count of 150/mm³, within the laboratory reference range. Soft-tissue ultrasonography demonstrated a subcutaneous collection measuring 24 × 17 mm. The patient received ceftriaxone for seven days without clinical improvement. Direct examination of purulent material showed no microorganisms, and culture was sterile. A deep skin and subcutaneous tissue biopsy revealed moderate fibrosis associated with a dense inflammatory infiltrate rich in eosinophils, with no evidence of malignancy. Based on the clinical and histopathological findings, EF was considered the most likely diagnosis. Systemic corticosteroid therapy was initiated at 1 mg/kg/day, resulting in progressive clinical improvement. This case highlights the diagnostic challenge of localized EF in the absence of peripheral blood eosinophilia and in a patient with a history of recurrent abscesses and hypereosinophilic syndrome. EF should therefore be considered in the differential diagnosis of persistent indurated limb swelling, even when the presentation is localized and mimics an infectious process.

## Introduction

Eosinophilic fasciitis (EF), also known as Shulman’s disease, is a rare inflammatory and fibrosing connective tissue disorder first described by Shulman in 1974. It is characterized by inflammation and thickening of the fascia, with progressive fibrosis of the subcutaneous tissues, leading to skin thickening and induration, predominantly affecting the extremities [1,2]. Clinically, EF typically begins with edema and painful swelling of the limbs that may progressively evolve into induration and sclerosis, sometimes resulting in functional impairment [1,2]. Peripheral blood eosinophilia is frequently observed, particularly during the early stages of the disease, but it is not a constant finding and its absence does not exclude the diagnosis [1-4]. Diagnosis is based on a combination of clinical, laboratory, imaging, and histopathological findings. Full-thickness skin-to-fascia biopsy is considered the reference diagnostic procedure, demonstrating inflammatory and fibrotic changes involving the fascia [1,3,4].

Although the precise pathophysiological mechanisms of EF remain incompletely understood, current evidence supports an immune-mediated process involving eosinophil activation, T-cell responses, and the release of inflammatory and profibrotic mediators. These mechanisms may contribute to fibroblast activation and progressive extracellular matrix deposition, ultimately leading to fascial fibrosis [4]. Several potential triggering factors have been reported, including trauma, strenuous physical activity, and infections, although no single etiological mechanism has been established [1-4].

The prevalence of EF remains poorly defined because of its rarity and probable underdiagnosis. Available evidence consists mainly of case reports and small case series. The disease predominantly affects adults, although pediatric cases have also been described [5-8]. Its clinical presentation may overlap with several infectious, inflammatory, eosinophilic, and sclerosing disorders, which may complicate diagnosis, particularly in atypical or localized presentations.

Imaging can provide complementary information in patients with suspected EF. Magnetic resonance imaging (MRI) may help demonstrate fascial inflammation and thickening, assess the extent of fascial involvement, and complement clinical and histopathological evaluation [1,3,4]. Ultrasonography may also provide information regarding soft-tissue abnormalities, although it may not adequately characterize the extent of fascial involvement.

We report a case of EF in a 16-year-old patient with an atypical localized presentation following minor trauma. The study was conducted after obtaining informed consent from the patient and her parents.

## Case presentation

A 16-year-old female patient had a documented history of hypereosinophilic syndrome and moderate persistent asthma treated with a fixed combination of budesonide and formoterol. She also had a history of recurrent cutaneous abscesses involving the legs, thighs, and back, which had previously required surgical drainage. In the context of her previous clinical manifestations and underlying asthma, serum immunoglobulin E (IgE) levels had previously been assessed and were elevated at 1,000 IU/mL.

**Figure 1:**
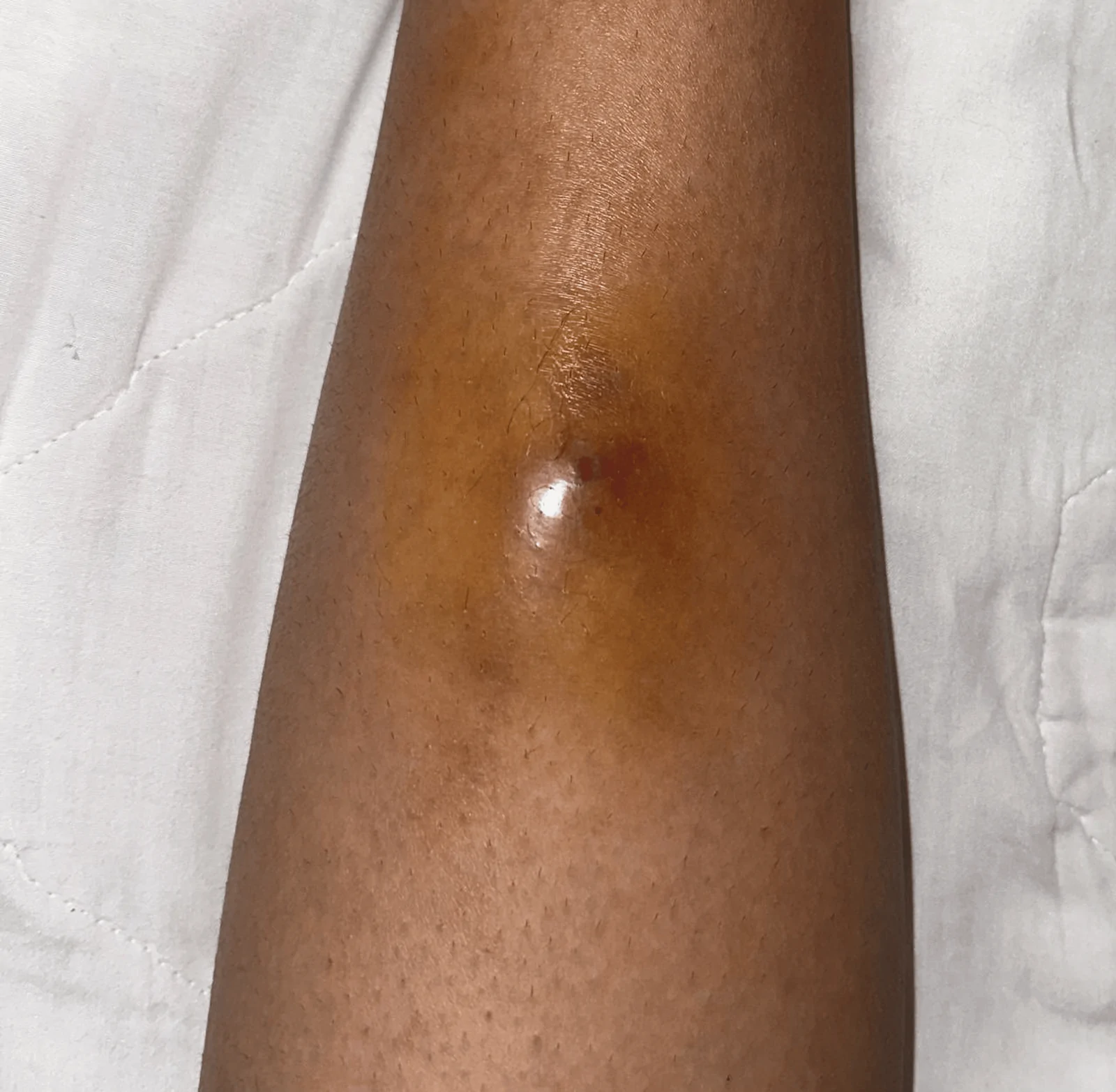
Initial clinical presentation showing an inflammatory swelling of the anterior aspect of the left leg, with erythema and skin tension

The patient presented with localized swelling of the left leg following minor trauma. The swelling appeared approximately one week after the trauma. The exact duration of the swelling before presentation was not documented in the available medical records. Clinical examination revealed a localized swelling measuring approximately 3 cm in diameter on the anterior aspect of the middle third of the left leg. The lesion was fluctuant and associated with local inflammatory signs, including erythema and warmth (Figure 1). Surgical scars were also noted on the upper third of the anterior aspect of the left leg, as well as on the right leg and thigh, consistent with her history of recurrent cutaneous abscesses.

Laboratory investigations showed a negative C-reactive protein level, a normal leukocyte count of 7,490/mm³, and an eosinophil count of 150/mm³, which was within the laboratory reference range (Table 1). Lymphocyte and neutrophil counts were also within their respective reference ranges.

**Table 1:** Laboratory investigations

Parameter	Value	Reference Range
CRP	Negative	< 5 mg/L
Leukocytes	7,490 /mm³	4,000 – 10,000 /mm³
Eosinophils	150 /mm³	0 – 500 /mm³
Lymphocytes	2,480 /mm³	1,000 – 4,000 /mm³
Neutrophils	4,260 /mm³	1,500 – 7,500 /mm³

**Figure 2:**
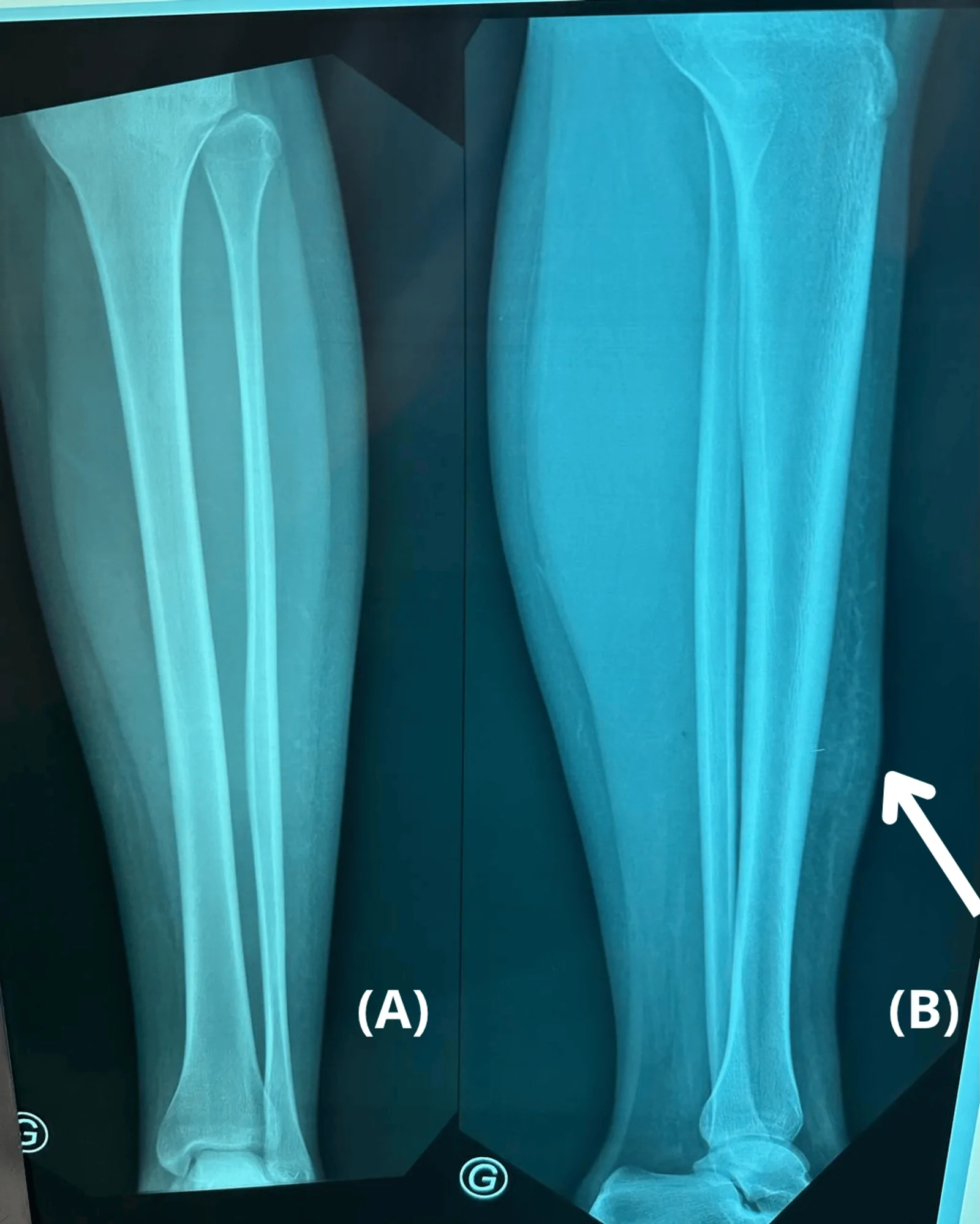
(A) Anteroposterior view of the left leg radiograph confirming the absence of osseous involvement. (B) Lateral view showing soft tissue swelling without bone abnormalities.

Soft-tissue ultrasonography revealed a subcutaneous collection measuring 24 × 17 mm on the anterior aspect of the middle third of the left leg. Plain radiography of the left leg demonstrated soft-tissue swelling without associated bone abnormalities (Figure 2). Magnetic resonance imaging was not performed.

Given the fluctuant appearance of the lesion, the ultrasonographic finding of a subcutaneous collection, and the patient's history of recurrent cutaneous abscesses, a recurrent abscess was initially considered. The patient received ceftriaxone for seven days without clinical improvement. A sample of the purulent material was obtained for microbiological analysis; direct examination showed no microorganisms, and the culture remained sterile. The absence of clinical improvement despite antibiotic therapy, together with the negative microbiological findings, negative C-reactive protein, and normal leukocyte count, made a bacterial abscess less likely.

A deep skin and subcutaneous tissue biopsy was subsequently performed and demonstrated moderate fibrosis associated with a dense inflammatory infiltrate rich in eosinophils. No evidence of malignancy was identified. Based on the available histopathological documentation, involvement of the fascia could not be confirmed retrospectively.

**Figure 3:**
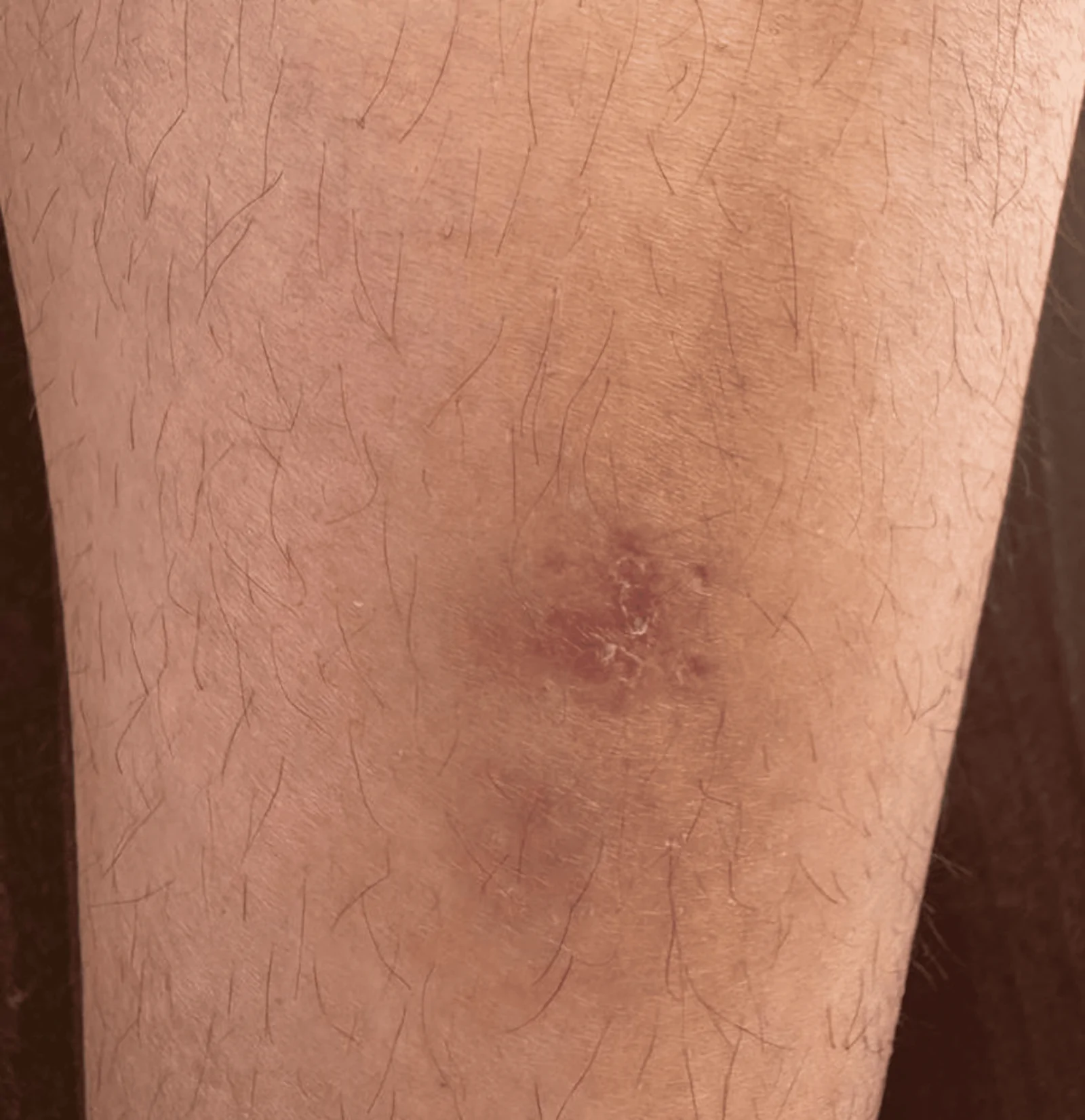
Follow-up clinical appearance showing a cicatricial lesion on the anterior aspect of the left leg

Based on the clinical presentation, the absence of convincing evidence of bacterial infection, and the histopathological findings showing fibrosis associated with a dense eosinophilic inflammatory infiltrate, EF was considered the most likely diagnosis. Systemic corticosteroid therapy was initiated at a dose of 1 mg/kg/day, resulting in progressive clinical improvement with early signs of lesion healing (Figure 3).

## Discussion

EF is a rare inflammatory and fibrosing connective tissue disorder that predominantly affects adults, although pediatric cases have also been reported [1-3,7,8]. In our case, EF was considered in a 16-year-old adolescent who presented with localized swelling of the left leg following minor trauma. This presentation differs from the more typical diffuse involvement of the extremities described in the literature. Pediatric cases may show heterogeneous clinical presentations, which can contribute to diagnostic delay, particularly when the disease presents atypically [5-9].

The localized nature of the lesion and the patient’s history of recurrent cutaneous abscesses initially raised the possibility of a recurrent abscess. This hypothesis was further supported by the fluctuant appearance of the lesion and the subcutaneous collection measuring 24 × 17 mm on ultrasonography. However, several findings subsequently argued against a typical bacterial abscess. The patient received ceftriaxone for seven days without clinical improvement. Direct examination of the purulent material revealed no microorganisms, and the culture was sterile. In addition, the C-reactive protein level was negative, and the leukocyte count was within the normal range. Although a sterile culture after antibiotic exposure cannot completely exclude infection, the lack of clinical response to antibiotic therapy, together with the microbiological and laboratory findings, made a recurrent bacterial abscess less likely.

Another important feature of this case was the absence of peripheral blood eosinophilia. The eosinophil count was 150/mm³, which was within the laboratory reference range. Peripheral eosinophilia is frequently associated with EF but is not consistently present and may therefore be absent at the time of diagnosis [1-4]. In the series reported by Wright et al., peripheral eosinophilia was documented in 58% of patients [2]. Therefore, the normal eosinophil count in our patient did not exclude EF. In this context, the diagnosis was not based on peripheral eosinophilia but rather on the combination of the clinical presentation and the histopathological finding of fibrosis associated with a dense inflammatory infiltrate rich in eosinophils.

The patient's history of hypereosinophilic syndrome (HES) represented an additional diagnostic challenge. HES was documented in her previous medical records, and she also had moderate persistent asthma and a previously elevated serum IgE level of 1,000 IU/mL. Given the overlap between eosinophilic disorders, the possibility that the tissue eosinophilic infiltration represented a manifestation of the underlying HES had to be considered. However, at the time of the present episode, the peripheral eosinophil count was within the normal range, and the clinical manifestation was localized to the left leg. The available medical documentation did not provide sufficient information to definitively exclude HES-related tissue involvement. Therefore, this possibility should be acknowledged as part of the diagnostic uncertainty rather than considered completely excluded.

The histopathological findings provided an important argument in favor of EF. The biopsy demonstrated moderate fibrosis associated with a dense inflammatory infiltrate rich in eosinophils. These findings are compatible with the inflammatory and fibrosing characteristics described in EF [1-4]. However, the available pathology documentation does not allow us to confirm retrospectively whether the fascia itself was included in the biopsy specimen. Full-thickness biopsy extending to the fascia is considered the reference diagnostic procedure for EF [1,3,4]. Consequently, the histopathological findings in our patient should be interpreted cautiously, and the diagnosis is best considered as the most likely diagnosis rather than an unequivocally confirmed diagnosis.

The absence of magnetic resonance imaging represents another limitation of the diagnostic workup. MRI was not performed in our patient, although it may provide complementary information regarding fascial involvement and the extent of inflammatory changes in EF [1,3,4]. In contrast, ultrasonography in our patient demonstrated a subcutaneous collection but did not establish the presence or extent of fascial involvement. Therefore, the absence of MRI limited the radiological assessment of the fascia and should be acknowledged when interpreting the diagnostic findings.

Other possible differential diagnoses included inflammatory or eosinophilic disorders that may produce localized swelling and tissue eosinophilia. In particular, eosinophilic cellulitis (Wells syndrome) may present with localized inflammatory swelling and therefore represented a relevant differential diagnosis in this case. The histopathological description available for our patient emphasized fibrosis associated with a dense eosinophilic inflammatory infiltrate, which favored the fibrosing process considered characteristic of EF. However, because the histopathological documentation was limited and fascial involvement could not be confirmed, Wells syndrome and other eosinophilic conditions cannot be considered definitively excluded.

Post-traumatic inflammatory reactions and other infectious causes were also considered because of the temporal relationship between the trauma and the onset of swelling. However, the negative inflammatory and microbiological findings and the lack of clinical response to ceftriaxone made a bacterial infectious process less likely. The temporal relationship with trauma may represent a possible triggering event, although it cannot establish causality.

Minor trauma has been described as a possible triggering factor for EF [1-4]. In our patient, the swelling appeared approximately one week after minor trauma, suggesting a possible temporal association. However, this association does not establish causality. The heterogeneous distribution of triggering factors reported in pediatric cases further suggests that trauma may act as a precipitating factor in susceptible individuals rather than being a necessary feature of the disease [7-9].

Treatment of EF is not standardized, but systemic corticosteroids are considered the mainstay of initial therapy [2,4]. Our patient received systemic corticosteroid therapy at a dose of 1 mg/kg/day and subsequently showed progressive clinical improvement with early signs of lesion healing. More severe or refractory disease may require additional immunosuppressive treatment, including methotrexate [2,4,6,7]. The favorable clinical response in our patient was consistent with the therapeutic response reported in previous pediatric cases [5-9], although response to corticosteroids alone cannot establish the diagnosis.

Overall, this case illustrates the diagnostic difficulty of a localized presentation of EF in an adolescent with a normal peripheral eosinophil count, a history of recurrent cutaneous abscesses, and a documented history of HES. The initial clinical and ultrasonographic findings made a recurrent abscess a reasonable diagnostic consideration, but the absence of improvement after seven days of ceftriaxone, sterile microbiological investigations, negative C-reactive protein, and normal leukocyte count argued against a typical bacterial infection. The combination of tissue fibrosis and a dense eosinophilic infiltrate subsequently favored EF. Nevertheless, the absence of MRI and the lack of documentation confirming fascial involvement on biopsy represent important limitations. Therefore, EF should be considered the most likely diagnosis in this case rather than an unequivocally confirmed diagnosis.

This case emphasizes the importance of considering EF in patients with persistent or indurated limb swelling, even in the absence of peripheral blood eosinophilia. Atypical localized presentations may mimic recurrent abscesses or other inflammatory disorders, and careful integration of clinical, microbiological, imaging, and histopathological findings is essential for reaching the most appropriate diagnosis.

Finally, the clinical course in our patient was marked by clinical improvement. While some reports describe a satisfactory outcome under treatment [6,8,9], others report persistent functional sequelae, particularly in severe or late-diagnosed cases [7]. This highlights the importance of early diagnosis as a key prognostic factor. Thus, although EF shares common clinical features, variations between studies may be explained by differences in disease stage at diagnosis, initial severity, and individual immune responses.

## Conclusions

Eosinophilic fasciitis is a rare disorder in children and may present with atypical, localized limb swelling that can mimic a recurrent abscess, particularly following minor trauma. In our patient, the normal peripheral eosinophil count did not exclude the diagnosis, while the absence of clinical response to antibiotic therapy, sterile microbiological investigations, and the presence of fibrosis with a dense eosinophilic inflammatory infiltrate supported eosinophilic fasciitis as the most likely diagnosis. However, the absence of MRI and the lack of documentation confirming fascial involvement on biopsy represent important limitations of the diagnostic work-up. Therefore, eosinophilic fasciitis should be considered in the differential diagnosis of persistent indurated limb swelling, even in the absence of peripheral eosinophilia. Early recognition and appropriate treatment, including systemic corticosteroid therapy when EF is considered the most likely diagnosis, may help prevent progression and functional complications.
